# Interventions designed to reduce implicit prejudices and implicit stereotypes in real world contexts: a systematic review

**DOI:** 10.1186/s40359-019-0299-7

**Published:** 2019-05-16

**Authors:** Chloë FitzGerald, Angela Martin, Delphine Berner, Samia Hurst

**Affiliations:** 1 iEH2 (Institute for Ethics, History and the Humanities), Faculty of Medicine, University of Geneva, Geneva, Switzerland; 2 Department of Philosophy, University of Fribourg, Fribourg, Switzerland

**Keywords:** Implicit prejudice, Implicit stereotype, Implicit bias, Unconscious bias, Interventions, Training, Professional ethics

## Abstract

**Background:**

Implicit biases are present in the general population and among professionals in various domains, where they can lead to discrimination. Many interventions are used to reduce implicit bias. However, uncertainties remain as to their effectiveness.

**Methods:**

We conducted a systematic review by searching ERIC, PUBMED and PSYCHINFO for peer-reviewed studies conducted on adults between May 2005 and April 2015, testing interventions designed to reduce implicit bias, with results measured using the Implicit Association Test (IAT) or sufficiently similar methods.

**Results:**

30 articles were identified as eligible. Some techniques, such as engaging with others’ perspective, appear unfruitful, at least in short term implicit bias reduction, while other techniques, such as exposure to counterstereotypical exemplars, are more promising. Robust data is lacking for many of these interventions.

**Conclusions:**

Caution is thus advised when it comes to programs aiming at reducing biases. This does not weaken the case for implementing widespread structural and institutional changes that are multiply justified.

**Electronic supplementary material:**

The online version of this article (10.1186/s40359-019-0299-7) contains supplementary material, which is available to authorized users.

## Background

A standard description of implicit biases is that they are unconscious and/or automatic mental associations made between the members of a social group (or individuals who share a particular characteristic) and one or more attributes (implicit stereotype) or a negative evaluation (implicit prejudice). Implicit prejudices are distinguished from implicit stereotypes in psychology: an implicit prejudice is supposedly a ‘hotter’ generic positive or negative feeling associated with a category, e.g. pleasant/white; an implicit stereotype involves a more belief-like association between a concept that is still valenced, but has fuller descriptive content, and a category, e.g. mentally agile/white. Although the distinction between implicit stereotypes and implicit prejudices is not as clear or necessarily as useful as much of the psychological literature assumes [1], it is important to track the distinction when analysing empirical findings because it can affect the results substantially. For example, Sabin and colleagues found that paediatricians demonstrated a weak implicit anti-black race prejudice (Cohen’s d = 0.41), but a moderate effect of implicit stereotyping, in which a white patient was more likely associated with medical compliance than a black patient (Cohen’s d = 0.60) [2].

The term implicit bias is typically used to refer to both implicit stereotypes and implicit prejudices and aims to capture what is most troubling for professionals: the possibility of biased judgement and of the resulting biased behaviour. Psychologists often define bias broadly; for instance, as ‘the negative evaluation of one group and its members relative to another’ [3]. However, on an alternative definition of bias, not all negative evaluations of groups would count as implicit biases because they are not troubling for our equity concerns. For instance, I might have a negative feeling associated with fans of heavy metal music – a negative implicit prejudice towards them. However, the fans of heavy metal music, as far as we are aware, are not a disadvantaged group, thus this implicit prejudice would not count as an implicit bias on this alternative definition. We thus stipulate that an implicit association (prejudice or stereotype) counts as implicit bias for our purposes only when it is likely to have a negative impact on an already disadvantaged group; e.g. if someone has an implicit stereotype associating young girls with dolls and caring behaviour, this would count as an implicit bias. It does not fit the psychologists’ definition above because it is not a negative evaluation per se, but it is an association that creates a certain image of girls and femininity that can prevent them from excelling in areas that are traditionally considered ‘masculine’ such as mathematics [4], and in which they already suffer discrimination. An example of an implicit prejudice that counts as a bias on our definition would be an association between negative feelings and homosexual couples - a negative implicit prejudice. This could disadvantage a group that already suffers discrimination and it thus qualifies as an implicit bias.

There has been much recent interest in studying the effects of implicit bias have on behaviour, particularly when that may lead to discrimination in significant areas of life, such as health care, law enforcement, employment, criminal justice, and education. Differing outcomes correlated with race, gender, sexual orientation, nationality, socio-economic status, or age, in these areas are likely to be partly the result of implicit biases, rather than or in addition to explicit prejudice or stereotyping. Given this fact, society has an interest in finding ways to reduce levels of implicit biases among the general population and among professionals who work in these areas in particular.

There is currently a growing awareness of implicit biases, particularly in the English-speaking world, and increasing attempts to counter them in professional settings. However, we found a lack of systematic evaluation of the evidence for the effectiveness of different interventions to reduce implicit bias.

In contrast to the recent study conducted by Forscher et al. [5], which used a technique new to psychology called network meta-analysis, and examined the effectiveness of procedures to change implicit bias, our focus was solely on the *reduction* of implicit social prejudice and implicit stereotypes, and only on those interventions that would be applicable in real world contexts and that were tested using the most widely employed implicit measure, the Implicit Association Test (IAT) and similar measures. Forscher et al.’s scope was wider because they investigated all changes in implicit biases of all kinds, admitted studies employing a variety of implicit measures, and did not restrict types of intervention.

Despite an unclear evidence base for their usefulness, interventions and training sessions to reduce implicit bias are being offered in the English-speaking world. Our review was partly prompted by this fact. Interventions that are not designed based on empirical evidence have the potential to do more harm than good. For instance, when people are told to avoid implicit stereotyping it can actually increase their biases [6, 7]. Ineffective training sessions may give participants and companies false confidence when in fact the training has had no ameliorative effect. False confidence in this area is particularly problematic because there is evidence that being asked to reflect on instances where one has behaved in an unbiased manner actually increases implicit bias, while reflecting on presumed failures to be unbiased reduces it [8].

We conducted a systematic review of studies measuring the effects of interventions to reduce implicit biases in adults as measured by the IAT. Interventions had to be fairly easily applicable to real life scenarios, such as workplace or healthcare settings. We concentrated solely on implicit biases because interventions that target explicit biases may leave implicit prejudices and stereotypes intact. Given the wide variety of interventions tested using different methods, a systematic review was more apt than a meta-analysis. This variety in the literature is what prompted Forscher et al. to use a novel form of meta-analysis, called ‘network meta-analysis’, which had never previously been used in psychology.

To this date, the most broadly recognized measure of implicit biases is the IAT. The IAT is usually administered as a computerized task where participants must categorize negatively and positively valenced words together with either images or words, e.g. white faces and black faces for a Race IAT. The tests must be performed as quickly as possible. The relative speed of association of black faces with positively-valenced words (and white faces and negatively-valenced words) is used as an indication of the level of anti-black bias [9].

Since its creation, the IAT has been subject to analysis and criticism as a measuring tool in the academic world [5, 10, 11] and, more recently, in the wider media [12, 13], where its utility as a predictor of real-world behaviour is questioned. Some valid criticisms of the IAT are against unwise uses of it or against interpretations of results obtained with it, rather than against the measure itself. Caution about how to use and interpret the IAT has been advised by its own creators, such as Brian Nosek, who in 2012 warned against using it as a tool to predict individual behaviour, for example [14]. The fact that it is does not have a high test-retest reliability in the same individual is widely known among researchers who use it. For that reason, it is not useful as a tool to label individuals e.g. as ‘an implicit sexist’ or to predict their individual behaviour. However, the creators of the IAT frequently use it as a tool to compare levels of implicit prejudice/implicit stereotype in different populations and see how this correlates with differences in behaviour [15].

The results of the IAT are highly context specific, as much research shows [16]. That does not mean that it has no validity or no connection to behaviour, just that we need more research to better understand exactly what it is measuring and how that relates to behavioural outcomes. Challenges are to be expected when trying to measure a construct that is outside conscious awareness. The connection between all measures of psychological attitudes and behaviour is complex, as is the case with self-report questionnaires, designed to measure explicit attitudes. In fact, implicit attitude tests partly came about as a result of the ineffectiveness of self-report measures to predict behaviour. Even if the most extreme criticisms of the IAT were true and the constructs it measured had very little effect on behaviour, we would expect a virtuous person who finds discrimination based on race abhorrent to be disturbed to discover that she automatically associates a historically oppressed race that still suffers discrimination with negative qualities. Professionals with integrity should thus be concerned to eliminate psychological associations that belie their moral principles.

## Methods

Our research question was: which interventions have been shown to reduce implicit bias in adults? ERIC, PUBMED, PSYCHINFO were searched for peer reviewed studies published in English between May 2005 and April 2015. Our full search strategies are included in the Additional file 1.

### Study eligibility

Studies were included if they were written in English, participants were either all adults (over 18) or the average age was over 18, and they were published in peer-reviewed journals. We excluded minors because we were interested in interventions that would be applicable in workplaces, thus on adults. The intervention had to be a controlled intentional process conducted with participants in an experimental setting, with the aim of reducing an implicit prejudice or implicit stereotype. We limited our research to social stereotypes and prejudices against people, as opposed to animals, inanimate objects, etc. Prejudices and stereotypes had to involve pre-existing associations thus excluding novel associations. They also had to be against a specific target thus excluding more generalized ‘outgroup prejudice’. An outgroup, in contrast to an ingroup, is any group to which a person does not feel that she belongs, a ‘they’ as opposed to a ‘we’. [17]

In an optimal experimental design, an implicit pre-test and post-test would be conducted on the same subjects in addition to the inclusion of a control group. However, since this is rarely found in the literature, we included articles where the effect was measured in comparison to a control group with similar characteristics. An advantage of a design using only a control group is that it eliminates any concern about a training effect occurring in participants between performing the IAT pre- and post-test.

The effect of the intervention had to be measured using a valid implicit measure before and after the intervention. In order for results to be comparable, we only included studies employing the most frequently used measure, the IAT, or a measure derived from or conceptually similar to it, such as the SC-IAT (Single Category Implicit Association Test), GNAT (Go/No-go Association Task, BIAT (Brief Implicit Association Test). Paper-based or computer versions of these tests were permitted. The IAT is the most widely used measure, and thus the most criticized and tested measure. We needed to select one implicit measure because different measures, such as affective priming, potentially measure different psychological constructs.

The intervention had to be applicable to real-world contexts and thus of a length and kind that enabled it to be easily implemented in different areas where implicit bias is a potential problem (e.g. medicine, general education, police force, legal professions and judiciary, human resources). The ease of implementation criterion is a matter of judgment, but comparisons can be made with similar types of training, such as sexual harassment training. If the intervention could be adapted to make a programme of similar length to that of current trainings typically provided in these areas, it was deemed suitable. This criterion ruled out observations drawn from natural settings that could potentially be used to develop interventions (e.g. correlations between increased contact with the outgroup and reduced bias). Many articles were excluded on this basis. It also ruled out long-term interventions involving considerable time and emotional commitment from participants. For instance, if an intervention had involved weekly attendance at a course over the course of a year (not simply changes in students’ curricula), we would have excluded it. As it happens, no interventions needed to be excluded for this reason.

We also excluded interventions that were too invasive in a person’s private life or over a person’s bodily autonomy, such as forcing people to make new friends, drink alcohol at work to reduce biases, or direct brain stimulation. There remains a grey zone when it comes to invasiveness that is open to cultural difference (e.g. whether being touched by a person of the outgroup is considered invasive).

The effectiveness of the intervention in reducing levels of implicit bias had to be initially tested within a maximum of one month from the intervention. This did not rule out further testing after this initial test. Since we were interested in interventions that reduce bias, we excluded interventions undertaken with the aim of increasing an implicit prejudice or stereotype.

### Study selection

**Fig. 1:**
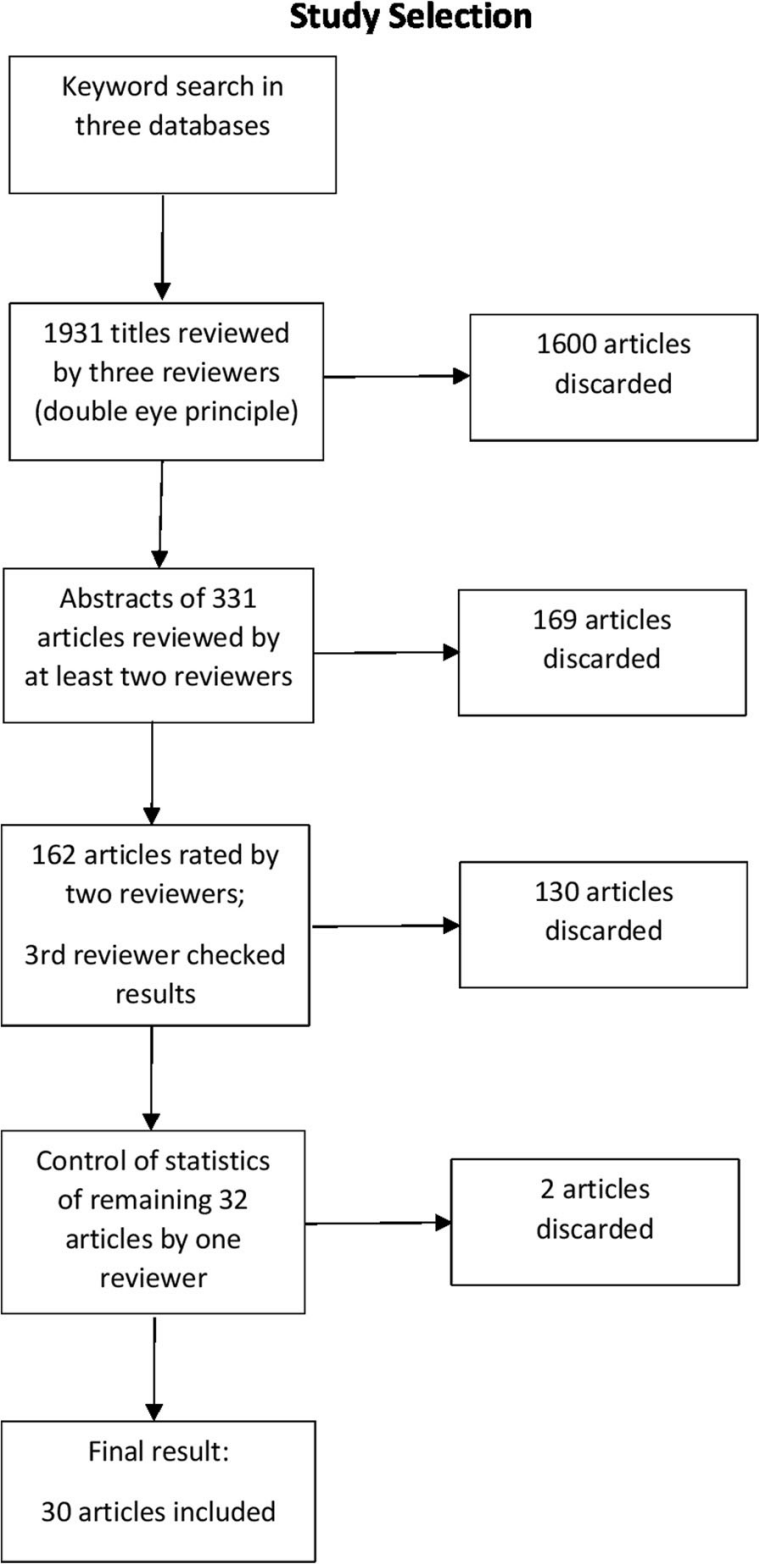
Study selection

The study selection process is illustrated in Fig. 1. Three reviewers, Angela Martin (AM), Chloë FitzGerald (CF) and Samia Hurst (SM), reviewed the 1931 titles resulting from the database searches. At least two of the three independently screened each title. Screening involved proposing the rejection of titles if there was a clear indication that the study did not fulfil our inclusion criteria. The titles that were agreed by both reviewers, or in case of uncertainty, by all three reviewers, after discussion to be ineligible according to the inclusion criteria were discarded (1600) and the abstracts of the remaining 331 articles were independently screened by at least two of the three reviewers. Abstracts that were agreed by both reviewers to be ineligible according to the inclusion criteria were discarded (169). When the ineligible abstracts were discarded, the remaining 162 articles were read and independently screened by at least two of the reviewers. After discussion, their decision on whether the article should be included was recorded and reviewed by the third reviewer who had not initially screened the article. SH reviewed the statistical analyses in the remaining 32 studies, which resulted in 2 articles being discarded due to lack of information about the statistical methods used. The final number of eligible articles was 30. However, one of the included articles [18] was in fact a competition organized to test different interventions created by different authors and thus involved 18 different interventions tested several times.^1^

### Data collection process

We based our inclusion criteria on the published results. If the data and methods used to calculate the results were not available in the article, we did not attempt to contact the authors to obtain this information. CF and AM independently extracted the data from the articles and each reviewed the other’s data when extraction was complete. All disagreements with the information extracted were resolved through discussion.

## Results

### Identified studies

As shown in Table 1, there are a total of 30 eligible articles. We have included the 18 interventions designed by different authors as part of a competition, all described in a single article [18], as separate entries to aid comprehension of the table, thus making a total of 47 different interventions tested. When there are slightly different eligible studies within one article, they are listed separately in the table only when the modifications produced a result that was different from the original study (in terms of being effective or ineffective at reducing bias).

**Table 1:** Articles included in systematic review

Type of intervention	Reference	Country	Bias	Type of Intervention	Effective
Engaging with others’ perspective, consciousness-raising or imagining contact with outgroup	Dermody, Jones, and Cumming 2013 [﻿19﻿]	Australia	Sexuality: male homosexual/male heterosexual	Imagined positive contact	No
	Turner and Crisp 2010 [20]	UK	Age: young/old	Imagined positive contact	Yes
			Religion: Muslim/non-Muslim		
	Rukavina et al. 2010 [21]	US	Obesity stereotype: fat/lazy versus thin/motivated	Classroom & service learning components, including perspective taking	No
	Swift et al. 2013 [22]	UK	Obesity	Educational films to induce empathy with outgroup	No
	Devine et al. 2012 [23]	US	Race: black/white	Multi-faceted prejudice habit-breaking intervention including perspective taking	Yes
	O’Brien et al. 2010 [24]	UK, US, Pakistan, New Zealand	Obesity	Tutorial on uncontrollable reasons for obesity (genes/environment)	Yes
	J.-L. Á. Castillo, Camara, and Eguizábal 2011 [25]	Spain	Race: Moroccan/ Native Spanish	Perspective taking / imagination	No
	Lehr: **Perspective Taking** [18]	US	Race: black/ white	Perspective taking / imagination	No
	Chen & Turner: **Imagining Interracial Contact** [18]	US	Race: black/ white	Imagined positive contact with outgroup and imagined negative contact with ingroup	No
	Schaefer: **Training Empathic Responding** [18]	US	Race: black/ white	Empathy training	No
	Park, Felix, and Lee 2007 [26﻿]	US	Race: Arab Muslims/black	Positive information about Arab-Muslims	Yes
Exposure to counterstereotypical exemplars	Joy-Gaba and Nosek 2010 [27]	US	Race: black/ white	Exposure to admired black exemplars and disliked white exemplars	Yes
	McGrane and White 2007 [28]	Australia	Racial: Asian/Anglo	Positive outgroup exemplars	Yes
	Columb and Plant 2011 [29]	US	Race: black/white	Obama as positive black exemplar	Yes
	Marini et al.: **Vivid Counterstereotypic Scenario** [18]	US	Race: black/ white	Vivid counterstereotypic scenario	Yes
	Teachman: **Practicing an IAT With Counterstereotypical Exemplars** [18]	US	Race: black / white	Practising IATs with counterstereotypical exemplars	Yes
	Frazier: **Shifting Group Boundaries Through Competition** [18]	US	Race: black/ white	Game where all teammates were positive and black and opponents all white and negative	Yes
	Lehr: **Shifting Group Affiliations Under Threat** [18]	US	Race: black / white	Study 2: Vivid post-apocalyptic scenario with positive black characters	No
				Study 3: Negative white characters added	Yes
	Kesebir: **Highlighting the Value of a Subgroup in Competition** [18]	US	Race: black/ white	Positive outgroup exemplars (famous basketball players)	No
Appeals to egalitarian values (activating egalitarian goals).	Blincoe and Harris 2009 [30]	US	Race: black/ white	Priming tolerance, respect or co-operation	Yes
	Clobert, Saroglou, and Hwang 2015 [31]	Belgium / Taiwan	Race: black/ white	Priming Buddhist concepts	Yes
			Religion: Christian/Muslim		
	L. G. Castillo et al. 2007 [32]	US	Race: black/white	Multicultural counseling classes	Yes
	Joy-Gaba: **Priming Feelings of Nonobjectivity** [18]	US	Race: black/ white	Priming feelings of non-objectivity	No
	Ho: **Priming an Egalitarian Mindset** [18]	US	Race: black/ white	Priming an egalitarian mindset	No
	Heiphetz: **Priming Multiculturalism** [18]	US	Race: black/ white	Priming multiculturalism	Yes
	Heiphetz: **Considering Racial Injustice** [18]	US	Race: black/ white	Considering racial injustice	No
	Hawkins: **Instilling a Sense of Common Humanity** [18]	US	Race: black/ white	Instilling a sense of common humanity	No
Identifying the self with the outgroup	Brannon and Walton 2013 [33]	US	Race: Latino/white	Cueing social connectedness with outgroup member	Yes
	Groom, Bailenson, and Nass 2009 [34]	US	Race: black/ white	Embodiment in black avatars	No
	Gündemir et al. 2014 [35]	The Netherlands	Race stereotype: Dutch/high status versus ethnic minority/low status; Dutch/leader versus ethnic minority/leader	Invoking a sense of identity with the outgroup	Yes
	Hall, Crisp, and Suen 2009 [36]	UK	Race: black/white	Experiment 1: Focusing on what ingroup and outgroup have in common	Yes
				Experiment 2: Listing overlapping subgroups of ingroup and outgroup	No
	Maister et al. 2013 [37]	UK, Hungary, the Netherlands	Race: black/ white	Multisensory stimulation to induce the feeling of ownership over a dark-skinned hand	Yes
	Peck et al. 2013 [38]	Spain, Italy, UK	Race: black/ white	Embodiment in black avatars	Yes
	Woodcock and Monteith 2013 [39]	US	Race: black/white	Ex. 1: Conditioning links between self and black	No
				Ex. 2: Conditioning links between self and black (replication and extension)	Yes
Evaluative conditioning	Calanchini et al. 2013 [40]	US	Race: black/ white	Affirm black-positive and white-negative picture pairings	Yes
	French et al. 2013 [41]	US	Race: Middle Eastern/white	Evaluative conditioning: Middle Eastern faces-positive and white faces -neutral	Yes
	Kawakami et al. 2007 [42]	US	Race: black/white	Approach/avoidance training	Yes
	Wojcik & Koleva: **Evaluative Conditioning** [18]	US	Race: black/ white	Study 1 & 2: Evaluative conditioning using IAT	No
				Study 3 & 4: Fewer trials	Yes
	Cerruti & Shin: **Evaluative Conditioning With the Go/No-Go Association Task** [18]	US	Race: black/ white	Study 1: Evaluative conditioning using GNAT	No
				Study 2–4: Fewer trials and minor modifications	Yes
Inducing emotion	Huntsinger, Sinclair, and Clore 2009 [43]	US	Race: black/white	Mood induction via music	Yes
	Huntsinger et al. 2010 [44]	US	Gender stereotype: men/leader versus women/supporter	Mood induction via music	Yes
	Haidt: **Inducing Moral Elevation** [18]	US	Race: black/white	Inducing moral elevation	No
	Lai, Haidt, and Nosek 2014 [45]	US	Sexuality: male homosexual/male heterosexual	Inducing moral elevation	Yes
Intentional strategies to overcome biases (override or suppress influence of biases)	Wallaert, Ward, and Mann 2010 [46]	US	Race: black/white	Told to avoid stereotyping on IAT	Yes
	Lai: **Using Implementation Intentions** [18]	US	Race: black/ white	Implementation intentions	Yes
	Lai: **Faking the IAT** [18]	US	Race: black/ white	Taught to try to fake responses on the IAT	Yes
Drugs	Terbeck et al. 2012 [47]	UK	Race: black/ white	Single oral dose of propanol (40 mg) in a randomised, double-blind, parallel group, placebo-controlled, design.	Yes

Titles in bold are interventions from the competition article [18]

We divided the interventions into 8 categories based on their psychological features. We used as our starting point modified versions of the 6 categories that had been created by the authors of the competition article of 17 interventions [18] and added two new categories. There are many different ways in which interventions can potentially be classified and we chose to base our categories on the ones already used in the competition article to facilitate discussion within the discipline. These categories are neither exhaustive nor completely exclusive. Our categories of intervention are:

1. Engaging with others’ perspective, consciousness-raising or imagining contact with outgroup – participants either imagine how the outgroup thinks and feels, are made aware of the way the outgroup is marginalised or given new information about the outgroup, or imagine having contact with the outgroup.
2. Identifying the self with the outgroup – participants perform tasks that lessen barriers between themselves and the outgroup.
3. Exposure to counterstereotypical exemplars – participants are exposed to exemplars that contradict the stereotype of the outgroup.
4. Appeals to egalitarian values – participants are encouraged to activate egalitarian goals or think about multiculturalism, co-operation or tolerance.
5. Evaluative conditioning – participants perform tasks to strengthen counterstereotypical associations.
6. Inducing emotion –emotions or moods are induced in participants
7. Intentional strategies to overcome biases – participants are instructed to implement strategies to override or suppress their biases.
8. Drugs – participants take a drug.

Effective interventions were those that showed a reduction in bias in the same individuals after the intervention in a pre−/post-test design, or in the group who underwent the intervention in a control group design. According to our criteria, the post-test had to be completed within a maximum of 1 month from the original intervention, but this did not rule out further tests at later dates.

The most effective categories were: intentional strategies to overcome biases (all 3 interventions were effective); exposure to counterstereotypical exemplars (7 out of 8 interventions had at least one effective instance); identifying the self with the outgroup (6 interventions out of 7 had at least one effective instance); evaluative conditioning (5 out of 5 interventions had at least one effective instance); and inducing emotion (3 out of 4 interventions were effective). The sole study in our drugs category was effective. The appeals to egalitarian values category had 4 interventions that were effective and 4 that were not. The largest category was engaging with others’ perspective, with 11 interventions, but a mere 4 of these were effective.

The number of studies in each category is small, thus strong conclusions cannot be drawn from these results. Patterns indicating clearly which methods were more successful as interventions were not visible. There is an indication that some directions may prove unfruitful, at least in short term bias reduction, such as engaging with others’ perspective, while exposure to counterstereotypical exemplars seems to be the most promising form of intervention, at least in the short term.

The country where studies were conducted was overwhelming the United States – US - (35 interventions), which explains why black/white race was the most examined bias in our review (34 interventions). There were 3 interventions aimed at Middle-Eastern/white bias and one each targeting Latino/white, Arab-Muslim/black and Asian/Anglo bias. Aside from race bias, 3 interventions were tested on weight bias, 2 on sexuality bias, 2 on religion bias, 1 on age bias and 1 on gender bias. 4 interventions were conducted in the United Kingdom (UK), 2 in Australia, 1 in Spain, 1 in the Netherlands, and 4 interventions were conducted in several different countries (including Belgium, Taiwan, Hungary, Italy, Pakistan and New Zealand). There was no clear pattern concerning whether some types of bias were more susceptible to interventions than others, given that the vast majority of articles in our review investigated black/white racial bias.

A majority of studies looked at implicit prejudice. However, 5 articles looked at implicit stereotypes as well as implicit prejudices in their interventions and 3 articles looked only at implicit stereotypes. Of these, only 3 interventions were effective at reducing stereotyping. The stereotypes investigated were the following: fat/lazy versus thin/motivated (3 articles); Dutch/high status versus ethnic minority/low status; Dutch/leader versus ethnic minority/leader (SC-IAT); men/leader versus women/supporter; men/science versus women/humanities; Spanish/active versus Moroccan/restful; white/mental versus black/physical.

### Limitations

#### Of specific studies

Although we judged all the studies in our review of sufficient quality to be included, the quality of the study design and statistical analysis employed varied greatly. One recurrent problem was the fact that there was often a lack of a proper statistical methods section and statistical tests used were instead reported in the results [26, 28, 38], or even in a footnote [46]. Some studies described their statistical methods only minimally [19, 25, 29, 31–33].

The paucity of empirically demonstrated effective interventions to reduce implicit bias and the pressure towards publishing positive results [48] is likely to tempt researchers to analyse data in a way that leads to positive results. The lack of statistical description suggests a risk of this.

An intervention tested by one study, rather than reducing implicit bias, actually increased it [34]. White participants who performed an intervention where they were embodied by a black avatar displayed greater implicit race bias than those who were embodied by a white avatar.

#### Of the field

Due to the interdisciplinarity of the subject and variety of fields from which articles proceeded (social psychology, medical ethics, health psychology, neuroscience, education, death studies, LGBT studies, gerontology, counselling, mental health, professional ethics, religious studies, disability studies, obesity studies) there was a lack of uniformity in the way that studies were described. In many cases, neither the titles nor the abstracts were very precise. They sometimes omitted to mention whether they tested implicit or explicit attitudes, a crucial piece of information e.g. [25, 41]. The distinction between implicit prejudice and implicit stereotype, which is important in the psychological literature, was also often blurred so that stereotype was cited in the title when the method described using an IAT to test implicit prejudice e.g. [41]. Methods and measures used were frequently omitted from the abstract, requiring the reader to read the article in full to gain this knowledge e.g. [31].

Many interventions were tested only on undergraduate psychology students, who are unlikely to be representative of the general population [49].

As is true in many areas, more replication studies are needed to confirm results. For example, two studies in our review tested a similar intervention, involving participants being embodied by a black avatar; while one found that the intervention actually increased implicit racial prejudice [34], the other found that it reduced it [38]. There were important differences between these two studies and the latter was not a replication study. All the interventions that are found to be effective in one study need to be replicated to provide confirmation.

There were some problems related to the indexing of articles: the keywords in PSYCHINFO and PUBMED in this field have changed frequently over the last few years because implicit bias is an emerging field of interest and study. Thus, indexing in databases was somewhat inconsistent making it difficult to capture all relevant articles with keywords. The fact that our search terms differed from those used by Forscher et al. [5], and that these differences were not all accounted for by differences in research question and inclusion criteria, is a sign of the problematic variations in terminology in the field.

The effects of interventions tend to be tested only over the short term. There were no longitudinal studies in our review. Even if short-term changes in biases are efficient, these changes will not be useful at providing practical solutions to discrimination unless they persist in the long term.

There is a risk that the sorts of stereotypes being studied are likely to be those that people are most aware of, and that stereotypes that are equally or more pernicious may be less visible and thus not be tested for. For instance, social class stereotypes can be hard to identify, especially given that they are not always clearly linked to economic status and that they may vary greatly from culture to culture. Furthermore, the sort of intervention tested is likely to be limited in scope to those that people think will be effective. For example, one philosopher has argued that many researchers are biased against certain effective techniques for reducing biases partly because they seem too mechanical [50]. The fact that such limited results have been found in the search for effective interventions may be caused by biases in researchers’ thinking.

While there are well-establish general publication biases in favour of positive publications, [48] we did not find this in our study as many published null results.

## Discussion

While several interventions aimed at reducing implicit biases had at least one instance of demonstrated effectiveness, the sample size was small and we were not able to identify reliable interventions for practical use. Thus, currently the evidence does not indicate a clear path to follow in bias reduction. Intentional strategies to overcome biases, evaluative conditioning, identifying the self with the outgroup, and exposure to counterstereotypical exemplars are categories that merit further research. Furthermore, caution is advised, as our review reveals that many interventions are ineffective; their use at present cannot be described as evidence-based.

As the authors of the competition study point out, the interventions that were successful in their competition had some features in common in reducing black/white race bias: the interventions that linked white people with negativity and black people with positivity were more successful than the ones that only linked black people with positivity; interventions where participants were highly involved, which means that they strongly identified with people in the scenarios that were used, were also successful [18]. Our category of identifying the self with the outgroup, which included several effective studies, includes this feature of high involvement.

There are similarities between our results and those from the recent network meta-analysis on change in implicit bias conducted by Forscher et al.: they found that procedures that associated sets of concepts, invoked goals or motivations, or taxed people’s mental resources produced the largest positive changes in implicit bias [5]; two of the categories that were most effective in our review, evaluative conditioning and counterstereotypical exemplars, involve associating sets of concepts, and interventions invoking goals or motivations would be included in our intentional strategies category, which also included effective interventions. Any confirmation between our review and that of Forscher et al. is of note, especially given that we used different search terms, research questions, and inclusion criteria. Forscher et al. also found that studies measuring interventions with the IAT rather than other implicit measures tended to produce larger changes in implicit bias. Overall, they found great variance in the effects of the interventions, which supports our conclusion that current interventions are unreliable. We do not yet know why interventions work in some circumstances and not in others and thus more fine-grained research is needed examining which factors cause an intervention to be effective.

So far, there has been very little research examining long-term changes in implicit attitudes and their effects on behaviour; the recent criticisms of the IAT mentioned in our introduction highlight this. Rather than invalidating the measure, they serve to show which directions future research with the IAT should go. In fact, in a follow-up study conducted by the same researchers as the competition study included in our review, interventions that had been demonstrated to be effective immediately were tested after delays of hours and days and none were found to be effective over these extended time periods [51].

To some extent, the ineffectiveness of interventions after a longer time period is to be expected. Implicit biases have been partly formed through repeated exposure to associations: their very presence hints at their being not only generated but also maintained by culture. Any counter-actions, even if effective immediately, would then themselves be rapidly countered since participants remain part of their culture from which they receive constant inputs. To tackle this, interventions may need to be repeated frequently or somehow be constructed so that they create durable changes in the habits of participants. More in-depth interventions where participants follow a whole course or interact frequently with the outgroup have been successful [51–53].

Unfortunately, this suggests that interventions of the type most desired by institutions to implement in training, i.e. short, one-shot sessions that can be completed and the requisite diversity boxes ticked, may simply be non-existent. If change is really to be produced, a commitment to more in-depth training is necessary.

In conducting the review, we were aware that interventions to reduce implicit biases were not sufficient to reduce prejudice in the public in general and in professionals in different fields on the long-term. These interventions should only form part of a bigger picture that addresses structural issues, social biases and may include more intensive training that aims to change the culture and society outside institutions in addition to within them [54]. Programmes in education to address the formation of stereotypes from much earlier on would be one way to effect longer term changes. In terms of addressing workplace culture, it may be worth reflecting on how culture changes are effected in institutions in other instances, such as in the case of medical error management in health care establishments. Affirmative action programmes that increase the numbers of women and minorities in leadership positions is one example of a policy with the potential to change the cultural inputs that foment implicit bias within a workplace.

Another approach that could be effective is to focus on reducing the impact of implicit bias on behaviour rather than reducing the bias itself. Organisational policies and procedures that are designed to increase equity will have an impact on all kinds of bias, including implicit bias. For example, collecting data that monitors equity, such as gender pay gaps, and addressing disparities, or reducing discretion in decision-making.

The majority of studies in our review only looked at effects of interventions on implicit prejudice, without investigating related implicit stereotypes. The lack of investigation into implicit stereotypes is troubling. Implicit prejudice is a measure of generic positive or negative implicit feelings, but it is likely that many behaviours that lead to micro-discriminations and inequalities are linked to specific and fine-grained stereotypes. This is particularly the case with gender stereotypes, as bias towards women is not typically linked to a generic negative feeling towards women, but towards women occupying certain roles that are not stereotypically ‘feminine’. For instance, one study found that only the implicit stereotype linking men with high status occupational roles and women with low status occupational roles predicted implicit and explicit prejudice towards women in authority. Other implicit stereotypes, linking women/home and men/career, or women/supportive and men/agential, lacked this predictive effect [55]. Only 8 of the articles in our review examined implicit stereotypes, but one of these found that an intervention that was effective at reducing implicit black/white race prejudice was not effective at reducing the implicit stereotype black/physical vs. white/mental [39]. Hence, it is not only important in the case of gender to investigate the effects of interventions on stereotypes as well as prejudice. The vast majority of studies on race prejudice seem to assume that it is the blanket positive/negative comparison of whites/blacks that needs to be addressed, but it could be the case that interventions will be more effective if they tackle more specific stereotypes.

A possible limitation of the review is that we included interventions that targeted different outgroups, and one may wonder whether interventions tested on one group are really applicable/effective to biases towards other groups. Indeed, if intervention X reduces the bias in group Y, it is by no means certain that same intervention is efficient to reduce bias against group Z. Implicit bias may well be a heterogeneous phenomenon [56]. On the other hand, an inefficient intervention X on group P may be efficient if tested for some other group or bias. Nonetheless, it is interesting to compare the types of intervention that are tested on different biases and to collect the evidence for interventions against different biases in one place. Often, researchers in a field interested in a particular bias, such as health professionals researching obesity, limit themselves to reading the literature on that bias and from their specific field and thus may overlook much evidence that could be relevant to their research. Furthermore, it may be that different biases require different types of intervention, but this can only be seen clearly if the different literatures are compared.

## Conclusions

Current data do not allow the identification of reliably effective interventions to reduce implicit biases. As our systematic review reveals, many interventions have no effect, or may even increase implicit biases. Caution is thus advised when it comes to programs aiming at reducing biases. Much more investigation into the long term effects of possible interventions is needed. The most problematic fine-grained implicit stereotypes need to be identified and a range of specifically-tailored interventions need to be designed to combat the whole gamut of prejudices that are problematic in our societies, not only targeting black/white race prejudice. More research needs to be conducted examining the conditions under which interventions will work and the factors that make them fail.

The fact that there is scarce evidence for particular bias-reducing techniques does not weaken the case for implementing widespread structural and institutional changes that are likely to reduce implicit biases, but that are justified for multiple reasons.

Our advice for future studies in this area can be summarized as follows:

- Investigate the effect of interventions on implicit stereotypes as well as implicit prejudices
- Use large sample sizes
- Pre-register study designs
- Use key words and titles that will span disciplines
- Include all relevant study parameters in the title and abstract
- Include all statistical analyses and data when publishing
- Include all the details of the study method
- Investigate the long term effects of interventions
- Investigate the effects of institutional/organizational changes on implicit biases
- Test interventions on a wide range of real workforces outside universities

## Additional file

Additional file 1: Full search strategies. (DOCX 15 kb)

## Abbreviations

AM: Angela Martin
BIAT: Brief Implicit Association Test
CF: Chloë FitzGerald
GNAT: Go/No-go Association Task
IAT: Implicit Association Test
SC-IAT: Single Category Implicit Association Test
SH: Samia Hurst
UK: United Kingdom
US: United States

## Footnotes

1 The title of the study lists 17 interventions, but the authors included a comparison condition, which makes a total of 18 interventions tested for our purposes.

## References

[1] Madva A, Brownstein M (2018). Stereotypes, prejudice, and the taxonomy of the implicit social mind. Noûs..

[2] Sabin JA, Rivara FP, Greenwald AG (2008). Physician implicit attitudes and stereotypes about race and quality of medical care. Med Care.

[3] Blair IV, Steiner JF, Havranek EP (2011). Unconscious (implicit) bias and health disparities: where do we go from here?. Perm J.

[4] Ambady N, Shih M, Kim A, Pittinsky TL (2001). Stereotype susceptibility in children: effects of identity activation on quantitative performance. Psychol Sci.

[5] Forscher PS, Lai CK, Axt J, Ebersole CR, Herman M, Devine PG, et al. A Meta-Analysis of Procedures to Change Implicit Measures. 2016. 10.31234/osf.io/dv8tu. 10.1037/pspa0000160 PMC6687518 31192631

[6] Payne BK, Lambert AJ, Jacoby LL (2002). Best laid plans: effects of goals on accessibility bias and cognitive control in race-based misperceptions of weapons. J Exp Soc Psychol.

[7] Galinsky AD, Moskowitz GB (2000). Perspective-taking: decreasing stereotype expression, stereotype accessibility, and in-group favoritism. J Pers Soc Psychol.

[8] Moskowitz GB, Li P (2011). Egalitarian goals trigger stereotype inhibition: a proactive form of stereotype control. J Exp Soc Psychol.

[9] Greenwald AG, McGhee DE, Schwartz JL (1998). Measuring individual differences in implicit cognition: the implicit association test. J Pers Soc Psychol.

[10] Oswald FL, Mitchell G, Blanton H, Jaccard J, Tetlock PE (2013). Predicting ethnic and racial discrimination: a metaanalysis of IAT criterion studies. J Pers Soc Psychol.

[11] De Houwer J. What are implicit measures and why are we using them. Handb Implicit Cogn Addict. 2006:11–28.

[12] Bartlett T. Can we really measure implicit bias? Maybe not. Chron High Educ. 2017.

[13] Singal J. Psychology’s favorite tool for measuring racism isn’t up to the job. N Y Mag. 2017.

[14] Nosek BA, Riskind RG (2012). Policy implications of implicit social cognition. Soc Issues Policy Rev.

[15] Greenwald AG, Banaji MR, Nosek BA (2015). Statistically small effects of the implicit association test can have societally large effects.

[16] Blair IV (2002). The malleability of automatic stereotypes and prejudice. Pers Soc Psychol Rev.

[17] Tajfel H (1970). Experiments in intergroup discrimination. Sci Am.

[18] Lai CK, Marini M, Lehr SA, Cerruti C, Shin J-EL, Joy-Gaba JA (2014). Reducing implicit racial preferences: I. a comparative investigation of 17 interventions. J Exp Psychol Gen.

[19] Dermody N, Jones MK, Cumming SR (2013). The failure of imagined contact in reducing explicit and implicit out-group prejudice toward male homosexuals. Curr Psychol.

[20] Turner RN, Crisp RJ (2010). Imagining intergroup contact reduces implicit prejudice. Br J Soc Psychol.

[21] Rukavina PB, Li W, Shen B, Sun H (2010). A service learning based project to change implicit and explicit bias toward obese individuals in kinesiology pre-professionals. Obes Facts.

[22] Swift JA, Tischler V, Markham S, Gunning I, Glazebrook C, Beer C (2013). Are anti-stigma films a useful strategy for reducing weight bias among trainee healthcare professionals? Results of a pilot randomized control trial. Obes Facts.

[23] Devine PG, Forscher PS, Austin AJ, Cox WT (2012). Long-term reduction in implicit race bias: A prejudice habitbreaking intervention. J Exp Soc Psychol.

[24] O’Brien KS, Puhl RM, Latner JD, Mir AS, Hunter JA (2010). Reducing Anti-Fat Prejudice in Preservice Health Students: A Randomized Trial. Obesity..

[25] Castillo J-LÁ, Camara CP, Eguizábal AJ (2011). Prejudice reduction in university programs for older adults. Educ Gerontol.

[26] Park J, Felix K, Lee G (2007). Implicit attitudes toward Arab-Muslims and the moderating effects of social information. Basic Appl Soc Psychol.

[27] Joy-Gaba JA, Nosek BA. The surprisingly limited malleability of implicit racial evaluations. Soc Psychol. 2010; [cited 2016 Jul 14]; Available from: http://econtent.hogrefe.com/doi/full/10.1027/1864-9335/a000020.

[28] McGrane JA, White FA (2007). Differences in Anglo and Asian Australians’ explicit and implicit prejudice and the attenuation of their implicit in-group bias. Asian J Soc Psychol.

[29] Columb C, Plant EA (2011). Revisiting the Obama effect: Exposure to Obama reduces implicit prejudice. J Exp Soc Psychol.

[30] Blincoe S, Harris MJ (2009). Prejudice reduction in white students: Comparing three conceptual approaches. J Divers High Educ.

[31] Clobert M, Saroglou V, Hwang K-K (2015). Buddhist concepts as implicitly reducing prejudice and increasing prosociality. Pers Soc Psychol Bull.

[32] Castillo LG, Brossart DF, Reyes CJ, Conoley CW, Phoummarath MJ (2007). The influence of multicultural training on perceived multicultural counseling competencies and implicit racial prejudice. J Multicult Couns Dev.

[33] Brannon TN, Walton GM (2013). Enacting cultural interests: How intergroup contact reduces prejudice by sparking interest in an out-group’s culture. Psychol Sci.

[34] Groom V, Bailenson JN, Nass C (2009). The influence of racial embodiment on racial bias in immersive virtual environments. Soc Influ.

[35] Gündemir S, Homan AC, de Dreu CK, van Vugt M (2014). Think leader, think white? Capturing and weakening an implicit pro-white leadership bias. PLoS One.

[36] Hall NR, Crisp RJ, Suen M (2009). Reducing implicit prejudice by blurring intergroup boundaries. Basic Appl Soc Psychol.

[37] Maister L, Sebanz N, Knoblich G, Tsakiris M (2013). Experiencing ownership over a dark-skinned body reduces implicit racial bias. Cognition..

[38] Peck TC, Seinfeld S, Aglioti SM, Slater M (2013). Putting yourself in the skin of a black avatar reduces implicit racial bias. Conscious Cogn.

[39] Woodcock A, Monteith MJ (2013). Forging links with the self to combat implicit bias. Group Process Intergroup Relat.

[40] Calanchini J, Gonsalkorale K, Sherman JW, Klauer KC (2013). Counter-prejudicial training reduces activation of biased associations and enhances response monitoring. Eur J Soc Psychol.

[41] French AR, Franz TM, Phelan LL, Blaine BE (2013). Reducing Muslim/Arab stereotypes through evaluative conditioning. J Soc Psychol.

[42] Kawakami K, Phills CE, Steele JR, Dovidio JF (2007). (Close) distance makes the heart grow fonder: Improving implicit racial attitudes and interracial interactions through approach behaviors. J Pers Soc Psychol.

[43] Huntsinger JR, Sinclair S, Clore GL (2009). Affective regulation of implicitly measured stereotypes and attitudes: Automatic and controlled processes. J Exp Soc Psychol.

[44] Huntsinger JR, Sinclair S, Dunn E, Clore GL (2010). Affective regulation of stereotype activation: It’s the (accessible) thought that counts. Pers Soc Psychol Bull.

[45] Lai CK, Haidt J, Nosek BA (2014). Moral elevation reduces prejudice against gay men. Cognit Emot.

[46] Wallaert M, Ward A, Mann T. Explicit Control of Implicit Responses. Soc Psychol. 2010. 10.1027/1864-9335/a000022 PMC3137766 21769299

[47] Terbeck S, Kahane G, McTavish S, Savulescu J, Cowen PJ, Hewstone M (2012). Propranolol reduces implicit negative racial bias. Psychopharmacology (Berl).

[48] Simmons JP, Nelson LD, Simonsohn U (2011). False-positive psychology: Undisclosed flexibility in data collection and analysis allows presenting anything as significant. Psychol Sci.

[49] Henrich J, Heine SJ, Norenzayan A (2010). Most people are not WEIRD. Nature..

[50] Madva A. Biased against Debiasing: On the Role of (Institutionally Sponsored) Self-Transformation in the Struggle against Prejuice. Open Access J Philos. 2017;4.

[51] Lai CK, Skinner AL, Cooley E, Murrar S, Brauer M, Devos T (2016). Reducing implicit racial preferences: II. Intervention effectiveness across time. J Exp Psychol Gen.

[52] Rudman LA, Ashmore RD, Gary ML (2001). “Unlearning” automatic biases: the malleability of implicit prejudice and stereotypes. J Pers Soc Psychol.

[53] Shook NJ, Fazio RH (2008). Interracial roommate relationships: An experimental field test of the contact hypothesis. Psychol Sci.

[54] Russell CA (2016). Questions of Race in Bioethics: Deceit, Disregard, Disparity, and the Work of Decentering. Philos Compass.

[55] Rudman LA, Kilianski SE (2000). Implicit and explicit attitudes toward female authority. Pers Soc Psychol Bull.

[56] Holroyd J, Sweetman J. The Heterogeneity of Implicit Bias. In: Brownstein M, Saul J, editors. Implicit Bias and Philosophy, Volume 1: Metaphysics and Epistemology: Oxford University Press; 2016. p. 80–103.

