# Supplementary material for: Interventions designed to reduce implicit prejudices and implicit stereotypes in real world contexts: a systematic review
Source: BMC Psychol. 2019 May 16;7:29. doi: 10.1186/s40359-019-0299-7 (PMC6524213; doi:10.1186/s40359-019-0299-7)
Supplement: Supplementary file 1 — Full search strategies. (DOCX 15 kb) [file 40359_2019_299_MOESM1_ESM.docx]

**For all databases :**

- **Dates: from 00:00 May 1st 2005 to 23.59 April 30th 2015**
- **English language selected**
- **Peer-reviewed articles selected**

**PUBMED**

Search terms:

*("prejudice"[MeSH Terms] OR "prejudice"[TW] OR "stereotyping"[MeSH Terms] OR “stereotype*”[TW])*

*AND (reduce[TW] OR reducing[TW] OR decrease[TW] OR decreased[TW] OR decreasing[TW] OR increase[TW] OR increased[TW] OR modify[TW] OR abolish*[TW] OR mediat*[TW] OR diminish[TW] OR "contextual variations"[TW] OR modifiability[TW] OR malleability[TW] OR "prejudice-reduction"[TW] OR "anti-prejudice"[TW] OR "Automatism/prevention and control"[Mesh] OR "Prejudice/prevention and control"[Mesh] OR train[TW] OR training[TW] OR “intervention”[TW] or manipulation)*

*AND ("implicit racial attitudes"[TW] OR “racial bias”[TW] OR “racial attitudes”[TW] OR “automatic bias”[TW] OR “automatic attitudes” OR "implicit bias*"[TW] OR “implicit anti-fat attitudes” OR “implicit prejudice”[TW] OR “racial prejudice” OR “racial stereotype” OR “african americans”[MeSH Terms] OR “european continental ancestry group”[MeSH Terms] OR race[MeSH Terms] OR "arabs/psychology"[MeSH Terms] OR "homosexuality/psychology"[MeSH Terms] OR “ethnic groups”[MeSH Terms] OR "Obesity/etiology"[Mesh] OR "Obesity/psychology"[Mesh] OR "Disabled Persons/psychology"[Mesh] OR "Disabled Persons/statistics and numerical data"[Mesh] OR "Mental Disorders/psychology"[Mesh] OR "Mental Disorders/statistics and numerical data"[Mesh] OR “implicit negative attitude*”[TW])*

*AND ("study"[TW] OR "studies"[TW] OR “experiment”[TW] OR “experiments” [TW])*

*NOT (Embryos OR "Binge-Eating Disorder"[Mesh] OR "Breast Feeding" OR "Menopause" OR "Evoked Potentials” OR "Education, Dental"[Mesh] OR "Television"[Mesh] OR "Risk-Taking"[Mesh] OR "Unsafe Sex"[Mesh] OR "Cardiovascular Diseases"[Mesh] OR "Cardiovascular System"[Mesh] OR "Metabolic Syndrome X"[Mesh] OR "Advertising as Topic"[Mesh] OR "Epilepsy"[Mesh] OR "Erectile Dysfunction"[Mesh] OR "Vaginal Diseases"[Mesh] OR "Transsexualism"[Mesh] OR "Pregnancy"[Mesh] OR "Anxiety Disorders"[Mesh] OR "HIV"[Mesh] OR “Health Services, Indigenous/ethics”[Mesh] OR “Health Status Disparities”[Mesh] OR “Interviews as Topic”[Mesh] OR “*[*Substance-Related Disorders/ethnology**](http://www.ncbi.nlm.nih.gov/pubmed/16809581)*”[Mesh] OR Narcotics OR “Healthcare Disparities*”[Mesh] OR Smoking OR "Sexually Transmitted Diseases"[Mesh] OR “Social Welfare”[Mesh] OR “Consumer Participation”[Mesh] OR “depressive disorder”[Mesh] OR "Acquired Immunodeficiency Syndrome"[Mesh] OR "Tuberculosis"[Mesh] OR “autism” OR “bulimia nervosa”[Mesh] OR “mammography” OR "Diabetes Mellitus"[Mesh])*

Notes:

- AIDS/ cancer research option (under Subject headings on the left) which is added automatically was diabled.
- Article type: nothing selected
- Species: Human

**PSYCHINFO (EbscoHost)**

Search Terms:

*(DE “prejudice” OR DE “stereotyped attitudes” OR DE “attitude change” OR DE “racial and ethnic attitudes” OR DE “racial and ethnic relations” OR DE “Stereotyped Behavior” OR DE “social groups”)*

*AND ((reduce OR reducing OR train OR training OR nonstereotypic OR decrease OR decreased OR decreasing OR increase OR increased OR improve OR mediate OR mediating OR modify OR lower OR abolish OR mediate OR control OR diminish OR diminished OR negative affect OR negative effect OR contextual variations OR modifiability OR malleability OR prejudice-reduction OR anti-prejudice)*

*AND (bias OR “implicit bias” OR “unconscious bias” OR “implicit “outgroup” bias” OR “stereotypic bias” OR “negative racial bias” OR “implicit racial bias” OR “intergroup bias” OR prejudice OR “implicit prejudice” OR “implicit negative attitude” OR “implicit negative attitudes” OR “implicit racial attitudes” OR “automatic attitude” OR “negative affect” OR prejudice OR “racial prejudice” OR stereotype OR “stereotypic responding” OR discrimination OR “automatic stereotyping” OR hiring OR recruitment))*

*AND (DE “intervention” OR interventions OR study OR studies OR experiment OR experiments OR research)*

*NOT (“major depression” OR psychometrics OR “visual perception” OR “animal learning” OR “evoked potentials” OR rats OR mice OR monkeys OR “motion perception” OR “prenatal development” OR symbolism OR “Cerebral Blood Flow” OR “Odor Discrimination” OR “Figure Ground Discrimination” OR “Breast Feeding” OR “Species Recognition” OR menopause OR “Neuroleptic Drugs” OR “Optical Aids” OR “Identity Formation” OR “achievement” OR “Lateral Dominance” OR “Chronic Pain” OR “Alternative Medicine” OR “Consumer Behavior” OR DE “AIDS prevention” OR Vocalization OR “Demographic Characteristics” OR “Acculturation” OR Phototherapy OR Food OR “Auditory Stimulation” OR Animal OR Epidemiology OR Amygdala OR DE “self-esteem” OR DE “memory” OR “Death Attitudes” OR “Eating Attitudes” OR “Childrearing Attitudes” OR “Client Attitudes” OR “Computer Attitudes” OR “Environmental Attitudes” OR “Family Planning Attitudes” OR Sports)*

Notes:

- Humans
- No Dissertations
- Empirical Studies

**ERIC (ProQuest, Uni Münster)**

Keywords:

(SU.exact("Social Bias") Or SU.exact("negative Attitudes") or SU.exact("social discrimination") or SU.exact("stereotypes"))

AND (reduce OR reduction OR reducing OR decrease OR decreasing OR increase OR increasing OR train OR training OR decrease OR decreased OR decreasing OR modifiability OR malleability OR prejudice-reduction OR anti-prejudice)

AND (Intervention OR study OR experiment)

AND (SU.exact("Attitude Change") OR SU.exact(“Consciousness raising”) OR SU.exact(“Program Effectiveness”) OR SU.exact(“Attitude Measures”) OR Effectiveness)

NOT (“couple therapy” or SU.exact(“marital satisfaction”) or SU.exact(“acquired immunodeficiency syndrome (AIDS)”) or SU.exact(“alcohol abuse”) OR SU.exact(“drinking”) OR SU.exact(“substance abuse”) OR SU.exact(“depression(psychology)”) OR “autism” OR SU.exact(“sexually transmitted diseases”) OR SU.exact(“photography”) or SU.exact(“family violence”))
